# Heart-Shaped Subhyaloid Haemorrhage in Valsalva Retinopathy With Successful Delayed Minimally Invasive Management

**DOI:** 10.7759/cureus.112587

**Published:** 2026-07-13

**Authors:** Shamim Choudhury, Adhikari Dissakaruna, Hallam Amos, Jai Shankar

**Affiliations:** 1 Emergency Department, Betsi Cadwaladr University Health Board, Wrexham, GBR; 2 Ophthalmology, Base Hospital Marawila, Marawila, LKA; 3 Ophthalmology, Aneurin Bevan University Health Board, Cardiff, GBR; 4 Ophthalmology, Wrexham Maelor Hospital, Wrexham, GBR

**Keywords:** diabetic retinopathy, expansile gas, intravitreal tpa, optical coherence tomography, premacular haemorrhage, preretinal haemorrhage, subhyaloid haemorrhage, valsalva retinopathy

## Abstract

Valsalva retinopathy is a recognised cause of acute painless visual loss resulting from a sudden increase in intrathoracic or intra-abdominal pressure, leading to subhyaloid haemorrhage. While most reported cases describe rounded or boat-shaped haemorrhages, atypical morphologies documented with multimodal imaging are infrequently reported. We describe a 45-year-old female with long-standing insulin-treated diabetes mellitus who developed sudden visual blurring following a violent coughing episode. Fundoscopic examination revealed a sharply demarcated, heart-shaped subhyaloid haemorrhage involving the macula, and optical coherence tomography confirmed a subhyaloid haemorrhage. Despite intervention occurring almost three weeks after symptom onset, intravitreal tissue plasminogen activator combined with expansile gas resulted in significant anatomical and functional recovery, with visual acuity improving from 6/36 to 6/9 and sustained improvement on follow-up. This case highlights the importance of recognising atypical haemorrhage morphology, accurately localising haemorrhage depth using optical coherence tomography, and individualising management. It also demonstrates that delayed minimally invasive intervention may achieve excellent visual outcomes, even in the presence of pre-existing retinal disease.

## Introduction

Valsalva retinopathy is a well-recognised cause of acute, painless visual loss resulting from a sudden rise in intrathoracic or intra-abdominal pressure. This leads to rupture of superficial retinal capillaries with subsequent accumulation of blood within the subhyaloid space [1]. Subhyaloid haemorrhage occurs when blood collects in the potential space between the posterior hyaloid face and the internal limiting membrane [2,3]. The macular region is particularly vulnerable because of relatively weak vitreoretinal adhesion, allowing blood to pool centrally and cause significant visual disturbance [4].

Although numerous cases of Valsalva retinopathy have been reported, most describe rounded or boat-shaped subhyaloid haemorrhages [3]. Reports of atypical haemorrhage morphology, particularly those supported by multimodal imaging and successful delayed interventional management, remain limited. We present a distinctive case of a heart-shaped subhyaloid haemorrhage following a Valsalva manoeuvre in a patient with underlying diabetic retinopathy, highlighting the diagnostic value of optical coherence tomography (OCT) and the importance of individualised management strategies.

## Case presentation

A 45-year-old female presented with a two-week history of sudden-onset painless visual blurring in the left eye following a violent coughing episode. She described perceiving a “floating heart” within her visual field. There was no history of ocular trauma or anticoagulant use. Her medical history was notable for long-standing insulin-treated type 2 diabetes mellitus complicated by diabetic retinopathy, diabetic nephropathy with proteinuria but preserved renal function, and treated hypertension. The patient was under regular ophthalmic follow-up for diabetic retinopathy.

**Figure 1:**
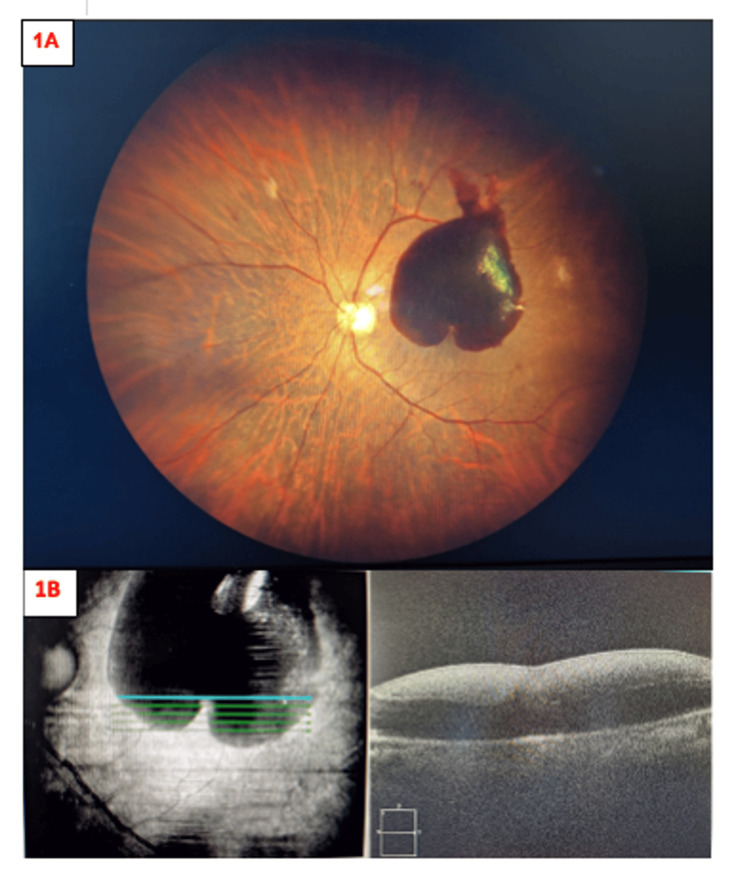
Multimodal imaging at presentation. (A) Colour fundus photograph of the left eye demonstrating a large (approximately four disc diameters), bright red, sharply demarcated, heart-shaped subhyaloid haemorrhage involving the macular region. (B) Optical coherence tomography at presentation demonstrating a subhyaloid haemorrhage overlying the fovea with preservation of the underlying retinal architecture.

On examination, best-corrected visual acuity was 6/9 in the right eye, improving to 6/6 with pinhole, and 6/36 in the left eye with no improvement on pinhole testing. The fellow eye demonstrated mild non-proliferative diabetic retinopathy without macular oedema. Fundoscopic examination of the left eye revealed a large (approximately four disc diameters), bright red, sharply demarcated, heart-shaped subhyaloid haemorrhage involving the macular region (Figure 1A). OCT demonstrated a subhyaloid haemorrhage overlying the fovea with preservation of the underlying retinal architecture (Figure 1B). Based on the clinical history and imaging findings, a diagnosis of Valsalva-associated subhyaloid haemorrhage was made.

Given the extent of macular involvement and degree of visual impairment, the patient underwent intravitreal injection of tissue plasminogen activator (tPA; alteplase) combined with an anti-vascular endothelial growth factor agent (ranibizumab biosimilar) and sulphur hexafluoride (SF₆) expansile gas under local anaesthesia. Treatment was initiated approximately three weeks after symptom onset.

**Figure 2:**
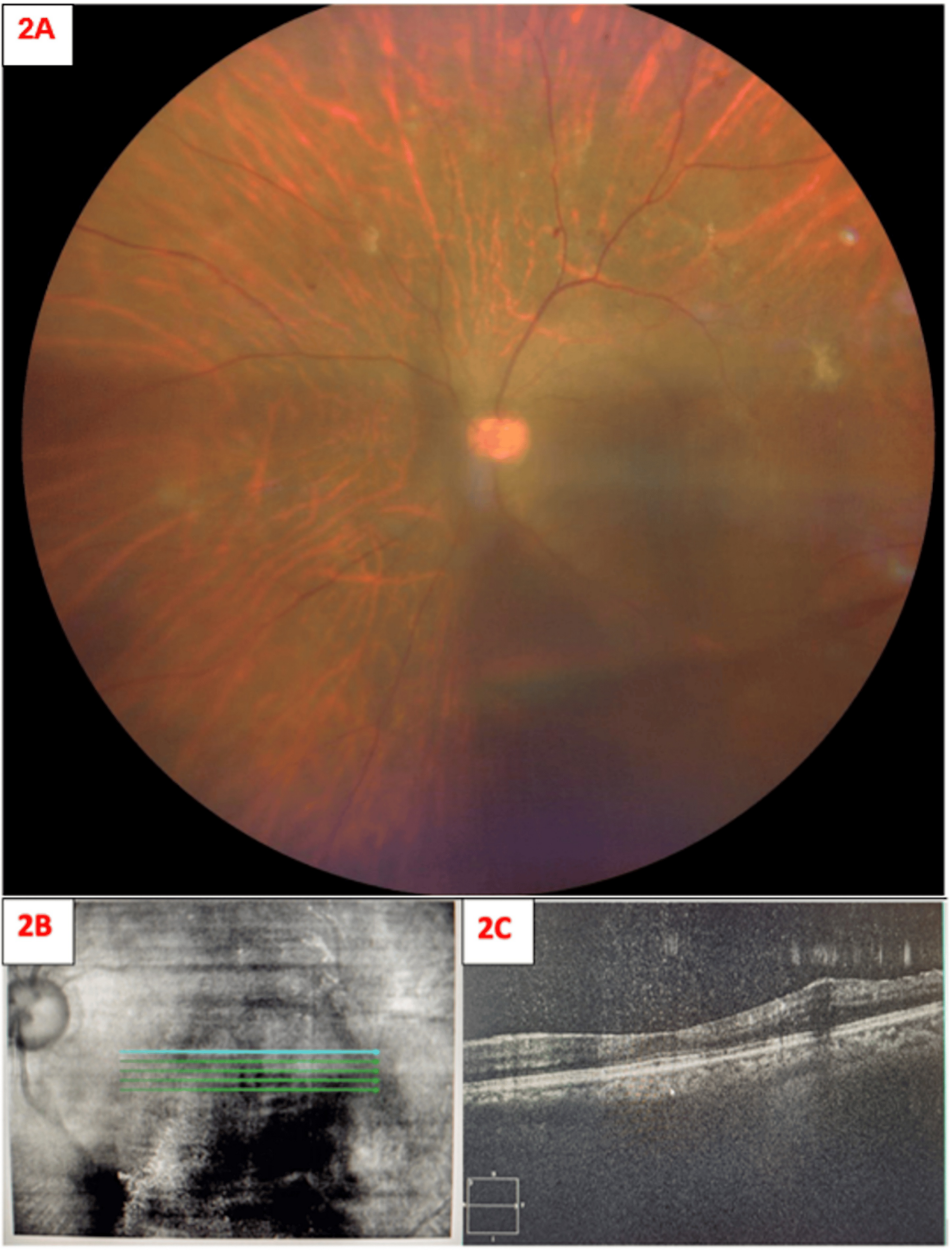
Multimodal imaging following intervention. (A) Colour fundus photograph of the left eye two weeks after treatment, demonstrating marked resolution of the subhyaloid haemorrhage. (B) Optical coherence tomography two days after intervention demonstrating early displacement of the subhyaloid haemorrhage. (C) Optical coherence tomography at two-week follow-up demonstrating near-complete resolution of the haemorrhage with restoration of normal macular contour.

At two days post-procedure, visual acuity in the left eye was 5/60. Fundoscopic examination demonstrated a slight reduction in the haemorrhage with approximately one-third gas fill, while OCT showed early displacement of the haemorrhage (Figure 2B). The patient was advised to continue face-down posturing for a further three days, followed by head-up positioning. At the two-week follow-up, visual acuity improved to 6/9 in the left eye. Fundus photography demonstrated marked resolution of the subhyaloid haemorrhage (Figure 2A), and OCT showed near-complete resolution with restoration of normal macular contour (Figure 2C). A small inferior vitreous haemorrhage was noted, with no evidence of retinal macroaneurysm or neovascularisation. No procedure-related complications occurred.

On subsequent follow-up, visual acuity in the left eye improved to 6/6 by approximately three months and remained stable on longer-term follow-up. The patient subsequently developed proliferative diabetic retinopathy requiring bilateral pan-retinal photocoagulation during follow-up. At final follow-up (approximately 17 months after treatment), visual acuity remained 6/9 in the left eye and 6/6 in the right eye, with no recurrence of subhyaloid haemorrhage.

## Discussion

The differential diagnosis of subhyaloid haemorrhage includes Valsalva retinopathy, Terson syndrome, retinal macroaneurysm, proliferative diabetic retinopathy, trauma, and retinal vein occlusion [2,5,6]. Valsalva retinopathy has also been reported following a wide range of activities associated with acute rises in intrathoracic or intra-abdominal pressure, including sexual intercourse, heavy weightlifting, general anaesthesia, and less obvious or habitual behaviours involving repeated bearing down [4,7-11]. This highlights the importance of a detailed clinical history and assessment of relevant ocular and systemic comorbidities when evaluating unexplained premacular haemorrhage. In this case, the clear temporal association with a violent coughing episode, characteristic imaging findings, and absence of alternative acute pathology supported a diagnosis of Valsalva-associated subhyaloid haemorrhage.

Subhyaloid haemorrhages are known to adopt characteristic configurations depending on the anatomical plane involved, as well as the effects of gravity, vitreoretinal adhesion, and clot organisation [2,12]. Commonly described morphologies include boat-shaped or dome-shaped haemorrhages within the subhyaloid space, crescentic or layered appearances in subhyaloid bleeding, and sharply demarcated premacular collections overlying the fovea [1,4,13-15]. Less frequently, atypical configurations have been reported, including heart-shaped appearances observed on OCT in the context of retinal haemorrhage [16]. These observations support the concept that anatomical confinement and biomechanical forces can give rise to unusual yet recognisable haemorrhage morphologies, as demonstrated in this case.

Accurate localisation of premacular haemorrhage is clinically important, as management strategies may differ depending on whether blood is situated in the subhyaloid or sub-internal limiting membrane (sub-ILM) plane [12,17]. Biomicroscopic examination alone may be insufficient to reliably distinguish these entities, particularly in the setting of dense haemorrhage [1,2]. Prior studies have emphasised the role of OCT in assisting haemorrhage localisation and guiding therapeutic decision-making, although image interpretation may be challenging when signal attenuation occurs due to overlying blood [7,12,18]. In this case, OCT was instrumental in confirming a subhyaloid location with preserved underlying retinal architecture, supporting a minimally invasive management approach.

Management options for Valsalva retinopathy include observation, neodymium-doped yttrium aluminium garnet (Nd:YAG) laser hyaloidotomy or membranotomy, pneumatic displacement with intravitreal gas with or without recombinant tPA, and pars plana vitrectomy [1,9,17,19-23]. In many cases, subhyaloid haemorrhages resolve spontaneously with excellent visual outcomes, and interventional treatment is not routinely required unless more rapid visual recovery is desired or the clinical course is complicated by persistent visual impairment or breakthrough vitreous haemorrhage [17,18].

Within this framework, intravitreal tPA combined with expansile gas has been proposed as an effective option in selected cases, with treatment decisions guided by the clinical context, OCT findings, symptom burden, and response to initial observation [17,18,24]. tPA is a recombinant fibrinolytic agent that promotes fibrinolysis by converting plasminogen to plasmin and is licensed for the treatment of thromboembolic conditions such as acute ischaemic stroke, myocardial infarction, and pulmonary embolism [25]. Its intravitreal use in ophthalmology represents an off-label application. While most published experience with intravitreal tPA relates to the management of submacular haemorrhage, its use in premacular or subhyaloid haemorrhage has also been described [26,27]. Small case series have reported successful resolution of premacular haemorrhage following intravitreal tPA administration, including studies in adults with diabetic retinopathy as well as reports in paediatric cases such as shaken baby syndrome, often in combination with expansile gas [28,29].

Larger retrospective studies have demonstrated that observation, laser intervention, and surgical approaches can all achieve excellent visual outcomes when treatment selection is guided by haemorrhage size, volume, duration, and functional visual impact [20,30]. Minimally invasive approaches, including intravitreal tPA combined with expansile gas, may facilitate clot lysis and pneumatic displacement while avoiding intraocular surgery [3,29,31].

In cases with coexistent proliferative retinopathy or ongoing vascular activity, intravitreal anti-vascular endothelial growth factor (anti-VEGF) therapy has been described as an adjunctive measure to reduce further bleeding risk, rather than as a primary modality for haemorrhage clearance [3,32,33]. This was relevant in the present case given the patient's background of diabetic retinopathy and the potential contribution of underlying retinal vascular disease to haemorrhage risk. Surgical intervention with pars plana vitrectomy remains an effective option for dense or persistent haemorrhages, providing immediate haemorrhage removal and definitive localisation, but carries inherent surgical risks, particularly in phakic patients [4,34].

Nd:YAG laser membranotomy may accelerate visual recovery in selected cases but is associated with recognised complications, including macular hole formation, retinal damage, and secondary membrane formation, particularly in sub-ILM haemorrhage. Nonetheless, retrospective studies have reported favourable outcomes following laser hyaloidotomy in Valsalva retinopathy and other causes of premacular subhyaloid haemorrhage, when patients are carefully selected [22,23,35,36]. Recurrence following laser treatment has been described and may necessitate escalation to pars plana vitrectomy, underscoring the importance of haemorrhage localisation, patient counselling, and activity modification following intervention [37].

In the present case, the haemorrhage occurred on a background of diabetic retinopathy, which may have increased capillary fragility and susceptibility to acute venous pressure changes during a Valsalva manoeuvre. Although spontaneous resolution is common, prolonged contact between blood products and the retinal surface may result in retinal toxicity and structural complications, while persistent central visual disturbance can significantly impair quality of life [1,2,8]. These considerations, together with the lack of early improvement and favourable OCT findings, supported intervention.

Notably, treatment was initiated almost three weeks after symptom onset, beyond commonly cited early intervention windows [17,36]. Despite this delay, significant anatomical and functional recovery was achieved. The small inferior vitreous haemorrhage observed following treatment may represent redistribution of liquefied blood after pneumatic displacement, as it was not associated with retinal macroaneurysm or neovascularisation and did not result in any adverse visual sequelae. In this case, anatomical and functional improvement was sustained on long-term follow-up (approximately 17 months) despite subsequent progression of diabetic retinopathy requiring bilateral pan-retinal photocoagulation. This outcome supports stepwise management approaches for Valsalva retinopathy, whereby observation is followed by selective intervention based on symptom persistence, haemorrhage characteristics, and OCT-confirmed anatomy.

Overall, this case highlights the importance of individualised management in Valsalva retinopathy. Careful assessment of haemorrhage morphology, anatomical localisation, symptom burden, and disease context is essential. OCT plays a pivotal role in confirming haemorrhage depth and guiding treatment selection, and minimally invasive strategies may provide substantial visual benefit even when intervention is delayed.

Learning points

Sudden painless visual loss following activities that increase intrathoracic or intra-abdominal pressure should raise suspicion for Valsalva-associated subhyaloid haemorrhage. Subhyaloid haemorrhages may adopt distinctive morphologies depending on the anatomical plane and vitreoretinal interface dynamics. Optical coherence tomography is valuable for determining the anatomical plane of haemorrhage and guiding management decisions. Even when intervention is delayed, minimally invasive approaches may achieve excellent and sustained anatomical and visual outcomes.

## Conclusions

This case describes an unusual heart-shaped subhyaloid haemorrhage associated with Valsalva retinopathy in a patient with diabetic retinopathy. It highlights the importance of accurate OCT-based localisation and demonstrates that selective minimally invasive intervention can achieve excellent and sustained anatomical and visual outcomes, even when treatment is initiated beyond traditionally cited early intervention windows.
